# Sex-Specific Association Between Zero-Calorie Beverage Consumption and Depressive Symptoms in Korean Adolescents: A Nationwide Cross-Sectional Study

**DOI:** 10.3390/nu18162610

**Published:** 2026-08-10

**Authors:** Yoosun Cho

**Affiliations:** Department of Family Medicine, Asan Medical Center, University of Ulsan College of Medicine, 88 Olympic-ro 43-gil, Songpa-gu, Seoul 05505, Republic of Korea; misslonghorn46@gmail.com; Tel.: +82-2-3010-3809; Fax: +82-2-3010-3815

**Keywords:** zero-calorie beverages, artificial sweeteners, depressive symptoms, adolescents, sex differences, cross-sectional study, Korea Youth Risk Behavior Web-based Survey

## Abstract

**Background/Objectives**: Zero-calorie beverages (ZCB) are increasingly consumed by adolescents, yet their relationship with mental health remains unclear, and whether any association differs by sex has not been established. We examined the association between ZCB consumption and depressive symptoms in a nationally representative sample of Korean adolescents, with sex as a prespecified effect modifier. **Methods**: This cross-sectional study analyzed 50,972 adolescents aged 12–18 years (50.9% male; mean age, 15.0 years) from the 2025 Korea Youth Risk Behavior Web-based Survey. Self-reported ZCB consumption during the previous 7 days and depressive symptoms during the previous 12 months were assessed. ZCB consumption was categorized as none, ≤2/week, 3–6/week, or ≥1/day. Survey-weighted logistic regression estimated adjusted odds ratios (aORs) with 95% confidence intervals (CIs), with non-consumers as the reference. Effect modification by sex was assessed using interaction terms. **Results**: Depressive symptoms were reported by 12,954 adolescents (25.4%). ZCB consumption was associated with higher odds of depressive symptoms across consumption categories (aOR for ≥1/day 1.76, 95% CI 1.45–2.13; *p* for trend < 0.001), after adjustment for sociodemographic and lifestyle covariates. The association appeared stronger among females than among males (aOR 1.14 [95% CI 1.11–1.18] vs. 1.05 [1.02–1.08]; *p* for interaction < 0.001), though significant in both sexes, and persisted after additional adjustment for co-consumed sugar-sweetened and high-caffeine beverages. **Conclusions:** In this nationally representative sample, daily ZCB consumption was associated with depressive symptoms, with a stronger association observed among females. Prospective studies are warranted to clarify temporality and underlying mechanisms.

## 1. Introduction

Depression is among the leading causes of disability during adolescence, with prevalence rising sharply across the second decade of life [1,2]. Globally, elevated depressive symptoms affect more than 30% of adolescents aged 10–19 years, and major depressive disorder increases through puberty—from approximately 1% in early adolescence to nearly 3% by late adolescence—with female adolescents consistently affected at higher rates than males [3,4]. This female predominance, which emerges during pubertal maturation, has been attributed to interactions between rising gonadal hormones and environmental exposures acting on developing brain circuitry [5,6,7]. In South Korea, depressive symptoms affect approximately one quarter to one third of adolescents, and the burden has increased since 2020, particularly among female students [8,9].

Concurrently, the diet of Korean adolescents has shifted toward greater consumption of ultra-processed foods and beverages [10]. Zero-calorie beverages (ZCBs), marketed as healthier alternatives to sugar-sweetened drinks, have become increasingly prevalent in the adolescent diet. In adults, several cohort studies have reported positive associations between artificially sweetened beverage consumption and depressive symptoms [11,12,13,14]. However, because ZCB consumption is not equivalent to exposure to a specific artificial sweetener, associations with ZCB intake should be interpreted in the context of broader beverage consumption patterns rather than individual sweetener effects.

Whether these adult observations generalize to adolescents remains unclear. Adolescence is a critical neurodevelopmental period during which prefrontal-limbic circuitry undergoes substantial reorganization and sex-specific vulnerability to mood disorders first emerges [15,16]. Consequently, the association between ZCB consumption and depressive symptoms may differ between girls and boys. In addition, most evidence linking diet and mental health in adolescents has been derived from Western populations, whereas East Asian adolescents, who differ in beverage consumption patterns and sociocultural context, remain relatively understudied [17,18]. Few studies, however, have specifically examined ZCB consumption in relation to depressive symptoms in adolescents, and evidence from Asian populations remains limited.

Extending this literature to an Asian adolescent population, this cross-sectional study aimed to evaluate the association between recent (past 7-day) ZCB consumption and past-year depressive symptoms in a nationally representative sample of Korean adolescents from the Korea Youth Risk Behavior Survey, and to examine sex as an effect modifier of this association.

## 2. Materials and Methods

### 2.1. Study Population and Design

We used cross-sectional data from the 2025 Korea Youth Risk Behavior Web-based Survey (KYRBS), an ongoing nationwide surveillance system established in 2005 to monitor health-risk behaviors among Korean middle- and high-school students. The KYRBS is administered annually by the Korea Disease Control and Prevention Agency (KDCA) in collaboration with the Ministry of Education, and raw data are publicly available [19]. A stratified multistage cluster sampling design generates a nationally representative sample, with response rates consistently exceeding 95%; all students in each sampled classroom complete an anonymous self-administered web-based questionnaire. Detailed survey methodology has been described previously [20]. Among 54,170 eligible participants, 3198 were excluded for missing data on exposure, outcomes, or covariates, leaving an analytic sample of 50,972 adolescents aged 12–18 years (Figure S1).

### 2.2. Measurements

Self-reported frequency of ZCB consumption during the preceding seven days was ascertained on a seven-point ordinal scale (1 = none; 7 = ≥3 times/day) and collapsed into four categories for analysis (none, ≤2/week, 3–6/week, ≥1/day). The outcome, depressive symptoms, was assessed with the standardized Korea Youth Risk Behavior Survey item asking whether, during the past 12 months, the respondent had ever felt so sad or hopeless almost every day for two weeks or more that they stopped doing some usual activities; a “yes” response was classified as the presence of depressive symptoms [9]. This single-item measure is a validated population-surveillance screen for clinically significant, functionally impairing depressive symptoms and does not constitute a clinical diagnosis of major depressive disorder [21].

Height and weight were self-reported, and body mass index (BMI) was calculated as weight (kg) divided by height squared (m^2^). Because adolescent body composition varies substantially by age and sex, weight status was classified using sex- and age-specific percentiles from the 2017 Korean National Growth Charts [22]: underweight (<5th percentile), normal weight (5th to <85th), and overweight or obese (≥85th), with reference to the WHO Growth Reference for school-aged children [23]. Sex was self-reported. Age was grouped as 12–13, 14–15, and 16–18 years, corresponding to early, middle, and late adolescence. School type was dichotomized into middle versus high school. Self-rated academic performance and household economic status were each collapsed into high, middle, and low categories.

Lifetime tobacco use was ascertained across conventional cigarettes, e-cigarettes, and heated tobacco products, with ever-smokers defined as those reporting lifetime use of any product. Lifetime alcohol use was self-reported, with ever-drinkers defined as those reporting any drinking beyond a small curiosity sip. Breakfast frequency was dichotomized as skipping (≤2 days/week) versus regular consumption. Physical activity was categorized as low (0–2 days/week), moderate (3–4), or high (≥5 days/week, meeting the WHO 2020 guideline) [24] based on the number of days with at least 60 min of moderate-to-vigorous activity. Daily smartphone use was derived as a weighted weekly average from separately reported weekday and weekend minutes. Sugar-sweetened and high-caffeine beverage frequencies were each categorized as less than weekly, weekly, or daily.

### 2.3. Statistical Analysis

All analyses accounted for the complex sampling design of the KYRBS, including sampling weights, strata, and primary sampling units, and were conducted using Stata version 19 (StataCorp, College Station, TX, USA). Baseline characteristics across categories of zero-calorie beverage (ZCB) consumption were compared using design-adjusted chi-squared tests for categorical variables and survey-weighted linear regression for continuous variables.

Survey-weighted logistic regression estimated adjusted odds ratios (aORs) with 95% CIs, with non-consumers as the reference. Model 1 was adjusted for sex, age, school type, household economic status, and academic performance. Model 2, the primary model, was further adjusted for BMI category, lifetime tobacco and alcohol use, physical activity, breakfast skipping, and daily smartphone use. Model 3 additionally adjusted for sugar-sweetened and high-caffeine beverage consumption and is presented alongside Model 2 as a more conservative analysis. Model 2 was specified as the primary model because sugar-sweetened and high-caffeine beverage consumption may represent either shared dietary-pattern confounders or intermediates on the pathway linking broader beverage-consumption patterns to depressive symptoms. *p* for trend was tested by entering the four-category exposure as an ordinal variable.

Effect modification by sex was evaluated using a categorical ZCB consumption × sex interaction term. Model-standardized predicted prevalences of depressive symptoms by sex and ZCB category were estimated from the fully adjusted (Model 2) survey-weighted logistic regression model using marginal standardization within each sex. These estimates represent the prevalence of depressive symptoms identified by the survey screening measure and not the incidence or risk of clinically diagnosed depression. Subgroup analyses were performed across categories of age, BMI, household economic status, academic performance, smartphone use tertile, physical activity, and breakfast skipping. Within each subgroup, aORs comparing daily ZCB consumption (≥1/day) with non-consumption were estimated using Model 2 covariates, excluding the stratifying variable; effect modification was assessed using interaction terms.

As sensitivity analyses, we assessed the robustness of the findings using an approximate servings-per-week exposure score (none = 0, ≤2 times/week = 1, 3–6 times/week = 4.5, and ≥1 time/day = 7 servings/week, with the highest category assigned its lower bound) and categorical beverage-consumption-by-sex interaction terms. Model-standardized prevalences were estimated as predictive margins from the multivariable survey-weighted logistic model, and adjusted prevalence differences and adjusted prevalence ratios were derived from these standardized prevalences; 95% confidence intervals were calculated using the delta method. Additive interaction between sex and daily zero-calorie beverage consumption was quantified using the relative excess risk due to interaction (RERI), calculated from adjusted prevalence ratios with male participants reporting no consumption as the reference group [25,26]. RERI was estimated from a single survey-weighted logistic model including sex, zero-calorie beverage consumption categories, and their interaction term.

Given the relatively small number of participants in the daily-consumption category, we repeated the sex-stratified analyses after combining the two highest categories (≥3 times/week) under both Model 2 and Model 3. All statistical tests were two-sided, with statistical significance defined as *p* < 0.05.

## 3. Results

### 3.1. Participant Characteristics

Of 50,972 Korean adolescents (50.9% male; mean age 15.0 years), 12,954 (25.4%) reported depressive symptoms. Most participants were non-consumers of ZCB (56.2%) or infrequent consumers (≤2/week, 37.5%); 5.0% reported intake 3–6/week and 1.2% ≥1/day. More frequent consumers were more likely to be male, older (16–18 years), and overweight or obese, and reported lower academic performance, higher prevalence of lifetime smoking and alcohol use, more frequent daily sugar-sweetened and high-caffeine beverage intake, and longer daily smartphone use (Table 1). Co-consumption of other beverages was substantial: among daily ZCB consumers, 50.7% also reported daily sugar-sweetened beverage intake and 24.3% daily high-caffeine beverage intake.

### 3.2. Association Between ZCB Consumption and Depressive Symptoms

ZCB consumption was associated with higher odds of depressive symptoms across consumption categories. In the fully adjusted model (Model 2), compared with non-consumers, aORs were 1.13 (95% CI 1.07–1.18; *p* < 0.001) for ≤2/week, 1.28 (1.16–1.42; *p* < 0.001) for 3–6/week, and 1.76 (1.45–2.13; *p* < 0.001) for ≥1/day, with a significant linear trend (*p* for trend < 0.001) (Table 2). Additional adjustment for sugar-sweetened and high-caffeine beverage consumption (Model 3) attenuated the associations but did not eliminate them. For consumption of ≥1/day versus none, the adjusted odds ratio was 1.32 (95% CI, 1.08–1.62) in Model 3, compared with 1.76 (1.45–2.13) in Model 2 (Table 2).

### 3.3. Sex-Differential Association

The association was modified by sex (*p* for interaction < 0.001) (Table 3). Among males, higher ZCB consumption categories were associated with increased odds of depressive symptoms, although category-specific estimates did not increase consistently (aORs 1.09 [1.01–1.16], 1.06 [0.92–1.22], 1.63 [1.28–2.07]; *p* for trend = 0.001); the estimate for 3–6/week was non-significant and not higher than that for ≤2/week. Among females, associations were stronger across higher consumption categories, with aORs of 1.16 (1.09–1.23), 1.65 (1.42–1.93), and 2.07 (1.54–2.79), respectively (*p* for trend < 0.001). However, these findings should be interpreted as category-specific associations rather than evidence of a biological dose–response relationship. The association for ≥1/day consumption remained statistically significant after additional adjustment for other beverage intake in both sexes—1.41 (1.02–1.95) in females and 1.32 (1.03–1.70) in males (versus 2.07 [1.54–2.79] and 1.63 [1.28–2.07] in Model 2, respectively) (Table 3).

**Figure 1:**
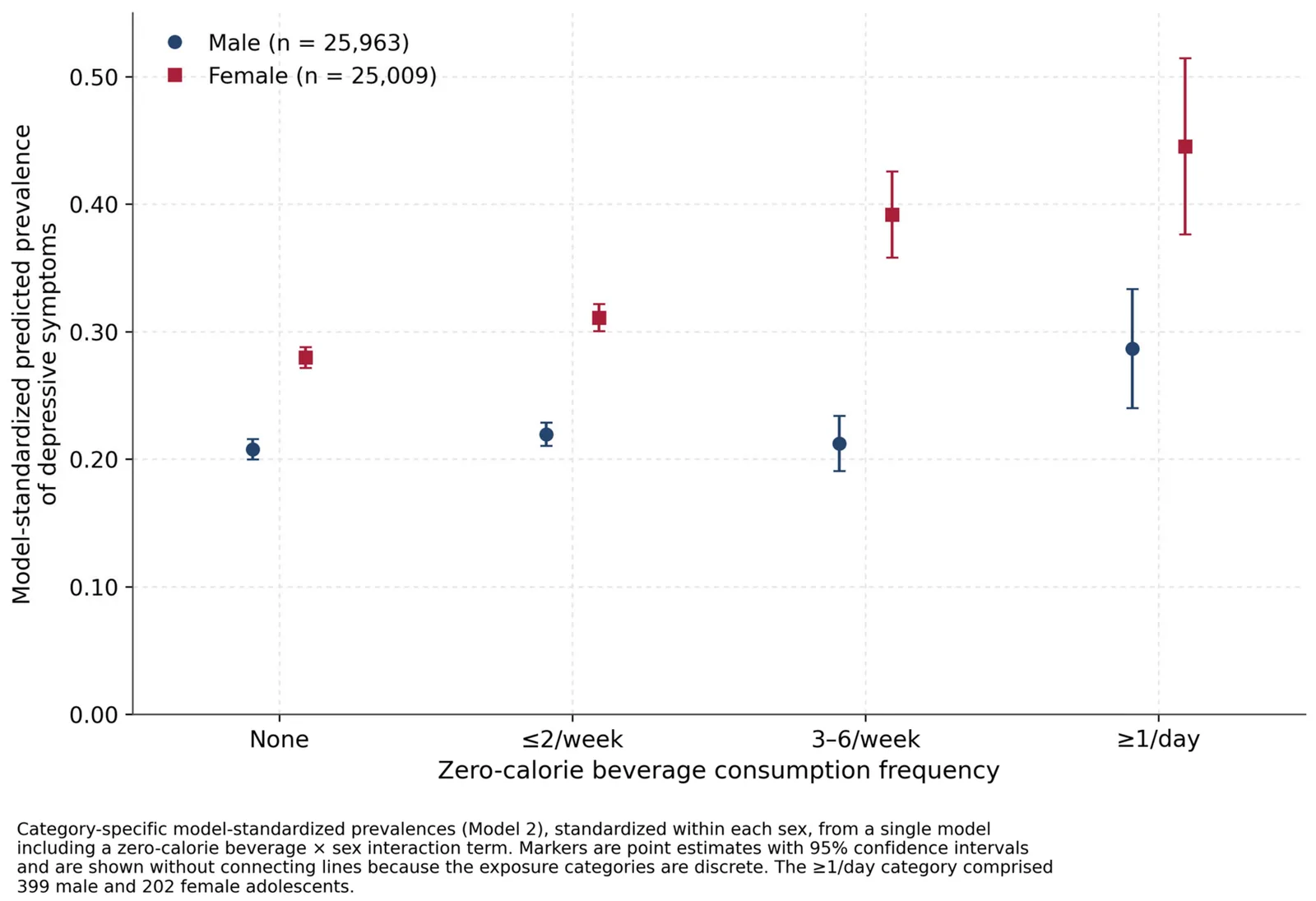
Model-standardized predicted prevalences of depressive symptoms according to zero-calorie beverage (ZCB) consumption category, by sex (Model 2), Korea Youth Risk Behavior Web-based Survey (KYRBS) 2025 (*n* = 50,972). Prevalences were estimated from a single survey-weighted logistic model including a ZCB-by-sex interaction term and standardized to the observed covariate distribution within each sex subgroup. Markers indicate category-specific point estimates, and error bars indicate 95% confidence intervals; estimates are shown without connecting lines because the exposure categories are discrete. Values represent the prevalence of depressive symptoms assessed by the survey screening item rather than the incidence of clinically diagnosed depression. The ≥1/day category comprised 399 male and 202 female adolescents.

The model-standardized predicted prevalence of depressive symptoms was highest among female adolescents reporting zero-calorie beverage consumption of ≥1/day (44.5%; Figure 1); because this category comprised relatively few participants (*n* = 202), the estimate should be interpreted with caution. These values reflect depressive symptoms identified by the survey screening measure rather than the risk or incidence of clinically diagnosed depression.

### 3.4. Subgroup Analyses

**Figure 2:**
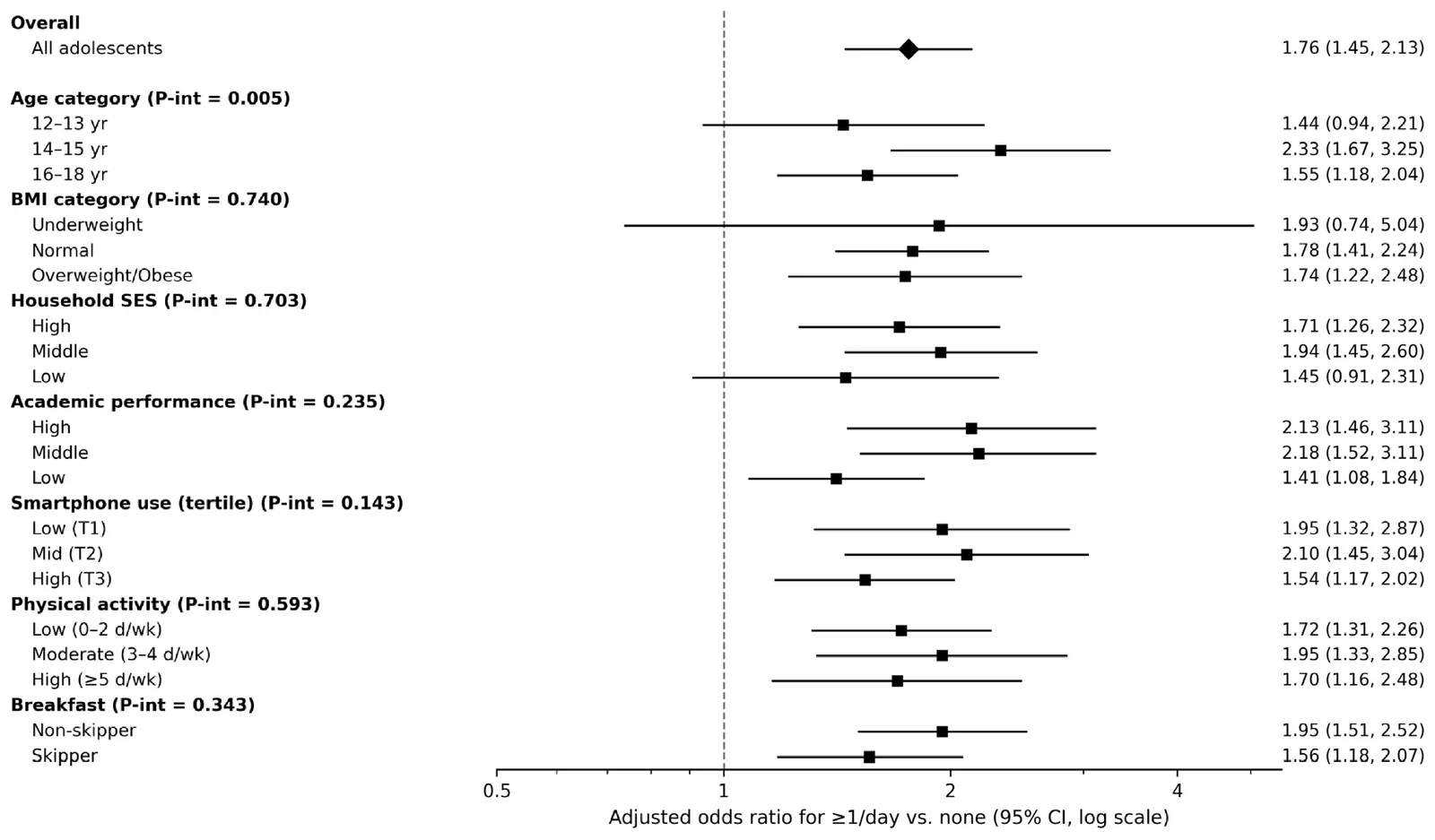
Subgroup consistency of the association between zero-calorie beverage (ZCB) consumption (≥1/day vs. non-consumption) and depressive symptoms (Model 2), KYRBS 2025 (*n* = 50,972). Squares are adjusted odds ratios with 95% confidence intervals for ≥1/day versus non-consumption within each subgroup (log scale); the diamond is the overall estimate. ZCB consumption was modeled as a categorical variable, and the figure should not be interpreted as implying a monotonic dose–response relationship. *p*-int denotes the Wald test for the exposure × subgroup interaction.

The association between ZCB consumption (≥1/day vs. non-consumption) and depressive symptoms was generally consistent across subgroups (overall adjusted OR 1.76, 95% CI 1.45–2.13; Figure 2). Effect modification was observed only for age (*p* for interaction = 0.005): the association was strongest among 14–15-year-olds (OR 2.33, 95% CI 1.67–3.25). No significant interactions were observed for BMI, household economic status, academic performance, smartphone use, physical activity, or breakfast skipping.

### 3.5. Sensitivity Analyses

On the standardized prevalence scale, the adjusted prevalence of depressive symptoms rose from 20.8% among males reporting no zero-calorie beverage consumption to 28.7% among those consuming at least one serving daily, and from 28.0% to 44.5% among females. The corresponding adjusted prevalence differences were +7.9 percentage points (95% CI, 3.1 to 12.7) in males and +16.6 percentage points (95% CI, 9.6 to 23.5) in females, and the adjusted prevalence ratios were 1.38 (95% CI, 1.15–1.61) and 1.59 (95% CI, 1.34–1.84), respectively (Table S1). The prevalence difference associated with daily consumption was 8.7 percentage points greater in females than in males (95% CI, 0.5 to 16.9), and the relative excess risk due to interaction was 0.42 (95% CI, 0.02 to 0.81; *p* = 0.038), indicating a significant positive additive interaction that was directionally consistent with the multiplicative interaction (*p* < 0.001).

Sensitivity analyses using alternative exposure coding showed similar sex-specific associations to the primary analyses, including analyses based on an approximate servings-per-week score and categorical exposure-by-sex interaction terms using indicator variables (Table S2). After combining the two highest categories, consumption of at least three times per week remained associated with depressive symptoms in both sexes, and the sex interaction remained significant (*p* < 0.001) (Table S3).

## 4. Discussion

In this nationally representative sample of 50,972 Korean adolescents, higher ZCB consumption was associated with greater odds of depressive symptoms after adjustment for sociodemographic and lifestyle covariates, and the association was stronger in female than in male adolescents (*p* for interaction < 0.001). Because depressive symptoms were common, odds ratios may overestimate relative associations; analyses using prevalence ratios and model-standardized prevalence differences yielded consistent findings. Given the cross-sectional design, these findings should be interpreted as sex differences in an observed association rather than as evidence of greater biological susceptibility among females. The model-standardized predicted prevalences were derived from a cross-sectional model using a population-based screening measure of depressive symptoms rather than a clinical diagnostic instrument, and therefore represent the prevalence of screening-defined depressive symptoms, not the incidence or risk of clinically diagnosed depression. In particular, the highest estimated value, observed among female daily consumers (44.5%), was derived from a relatively small subgroup (*n* = 202) and should not be interpreted as clinical risk.

Previous studies have linked soft drink consumption, including sugar-sweetened and artificially sweetened beverages, with depressive symptoms among adolescents, whereas evidence specifically addressing zero-calorie beverages remains limited. A systematic review and meta-analysis reported that both sugar-sweetened and artificially sweetened soft drink consumption were associated with depressive symptoms [27]. In US adolescents, high soft-drink consumption (≥3 times/day) was associated with higher odds of depressive symptoms (OR 1.61; 95% CI 1.42–1.81) [28]. A four-year prospective cohort of 1204 Chinese adolescents reported that those in the highest sugar-sweetened beverage tertile exhibited a 62% greater longitudinal increase in depressive symptoms [29], and a cross-sectional study of 33,523 Malaysian adolescents found that carbonated soft-drink intake was positively associated with depressive symptoms (adjusted OR 1.59; 95% CI 1.48–1.70) [30]. However, few population-based studies have specifically evaluated zero-calorie beverages in this age group, and evidence regarding potential sex differences remains limited.

In adults, large cohort studies have linked artificially sweetened beverage intake to an increased risk of depression [11,12,13,14], including a 23% higher incidence in the UK Biobank [11] and approximately 1.37-fold higher odds across extreme intake categories in the Nurses’ Health Study II [14]. Proposed mechanisms include alterations in the gut microbiome and downstream effects on neurobiological pathways involved in mood regulation [31,32]. The present findings extend this literature to adolescents by suggesting that higher ZCB consumption is associated with depressive symptoms in a large, nationally representative sample of Korean adolescents. The association appeared more pronounced among female than male adolescents, although confirmation from prospective studies is needed.

The stronger association among females was evident on the absolute as well as the relative scale (RERI, 0.42; 95% CI, 0.02 to 0.81), suggesting a greater excess burden of depressive symptoms associated with daily zero-calorie beverage consumption among female than among male adolescents. Several biological mechanisms may help explain this pattern. Adolescence is characterized by substantial pubertal hormonal changes and ongoing maturation of prefrontal-limbic and reward-related neural circuitry, coinciding with sex differences in depression [5,6,7,15]. Sex steroids influence serotonergic, dopaminergic, and hypothalamic-pituitary-adrenal axis function and may increase susceptibility to environmental exposures, including dietary factors, among female adolescents [33,34].

Experimental studies have also suggested that certain artificial sweeteners may alter gut microbial composition and neurobiological pathways involved in mood regulation through the gut-brain axis [31,32]. In addition, experimental models indicate that exposure to dietary sweeteners during adolescence may induce sex-dependent changes in affective behavior [35,36]. However, because the present study assessed the frequency of ZCB consumption rather than exposure to specific artificial sweeteners, and did not measure these biological mediators, these proposed mechanisms should be considered hypothesis-generating rather than directly supported by the present findings.

Behavioral and psychosocial factors may also contribute to the stronger association observed among female adolescents. Adolescents experiencing depressive symptoms may preferentially choose zero-calorie beverages as part of dieting attempts, weight-management strategies, or health-compensatory behaviors [37,38]. In addition, consumption of low-calorie beverages has been reported to be associated with dieting behaviors and parental weight-related concerns, suggesting that these beverages may serve as markers of weight-control practices rather than direct causes of adverse health outcomes [38]. Body-image concerns, dieting, and weight-control behaviors are more common among adolescent girls and are themselves associated with depressive symptoms [39]. These psychosocial factors may therefore contribute to both more frequent ZCB consumption and the stronger association observed among females. Consequently, the observed sex difference should not be interpreted as evidence that zero-calorie beverages have stronger biological effects among female adolescents but rather as a hypothesis-generating finding that may reflect behavioral and psychosocial factors.

Because sugar-sweetened and high-caffeine beverages may act as shared dietary-pattern confounders, potential intermediates in the association between broader beverage-consumption patterns and depressive symptoms, or both (Figure S2), their roles cannot be distinguished in this cross-sectional study. We therefore present both the more parsimonious Model 2 and the more conservatively adjusted Model 3. The attenuation of the association after additional adjustment is compatible with both shared dietary-pattern confounding and partial mediation through beverage co-consumption. Nevertheless, the association remained statistically significant after this conservative adjustment, suggesting that frequent zero-calorie beverage consumption is not merely a proxy for sugar-sweetened beverage intake. These findings should be interpreted cautiously because residual confounding and reverse causation cannot be excluded.

This study has several strengths. First, the KYRBS provides a large, nationally representative sample based on rigorous probability-based sampling, enabling population-level inference regarding beverage consumption and depressive symptoms among Korean adolescents. Second, we adjusted for a broad range of sociodemographic, anthropometric, and behavioral factors and conducted complementary analyses, including prevalence-scale analyses, additive interaction assessment, alternative exposure coding, and subgroup analyses, to examine the robustness of the findings. Third, by presenting both a parsimonious model and a model additionally adjusted for co-consumed sugar-sweetened and high-caffeine beverages, we were able to evaluate whether the observed association was consistent under different assumptions regarding these correlated beverage exposures.

Several limitations should also be considered. First, the cross-sectional design precludes inference about temporal relationships or causality. Because depressive symptoms were assessed over the previous 12 months whereas ZCB consumption referred to the previous 7 days, reverse causation cannot be excluded. Adolescents experiencing depressive symptoms may preferentially consume ZCBs because of dieting behaviors, body-image concerns, appetite changes, or health-compensatory behaviors. In addition, residual confounding by unmeasured psychosocial and behavioral factors cannot be ruled out. Prospective studies are needed to clarify the temporal relationship and causal direction. Second, zero-calorie beverage intake was assessed only as self-reported consumption frequency during the previous 7 days, without information on beverage volume, cumulative intake, brand, beverage formulation, caffeine content, or artificial sweetener subtype. This relatively coarse exposure assessment may have resulted in exposure misclassification. Because there is no strong reason to expect reporting accuracy to differ according to depressive symptom status, any resulting misclassification is likely to have been predominantly non-differential and would therefore tend to attenuate the observed associations toward the null. Accordingly, our findings should not be interpreted as evidence for dose-dependent or sweetener-specific associations. Third, depressive symptoms were ascertained with a single dichotomous self-report item rather than a validated multi-item instrument such as the PHQ-9. Although this item is consistently used in the KYRBS and other epidemiologic studies [8,9,21,40,41] and captures functionally impairing symptoms, any misclassification is also likely to be non-differential with respect to beverage intake and to bias estimates toward the null [42], although differential reporting by depressive-symptom status cannot be completely excluded. Fourth, information was lacking on family psychiatric history, menstrual-cycle phase, and pubertal development (Tanner stage). These factors may be associated with depressive symptoms and other health-related behaviors and could therefore have contributed to residual confounding, particularly in the sex-specific analyses. Fifth, the highest consumption category included relatively few participants, resulting in less precise estimates. However, a sensitivity analysis combining the two highest categories yielded findings consistent with the primary analysis, suggesting that the overall results were not materially influenced by the relatively small number of daily consumers (Table S3). Nevertheless, confirmation in future studies including larger numbers of frequent consumers is warranted. Finally, generalization of our findings beyond Korean adolescents should be undertaken with caution. In the present study, over 40% Korean adolescents reported consuming zero-calorie beverages at least once per week, with only 1.2% reporting daily consumption. Cross-national surveys have demonstrated considerable variation in diet soft drink consumption among European adolescents [43], while in the United States, 25.1% of children and adolescents reported any consumption of low-calorie sweetened beverages during 2009–2012 [44]. However, beverage consumption among Korean youth has increased substantially over the past two decades, suggesting a rapidly evolving dietary landscape despite currently lower absolute prevalence compared to Western countries [45]. Differences in beverage formulations, patterns of artificial sweetener use, dietary behaviors, and cultural norms surrounding body image and mental health may further influence both exposure distributions and the observed associations. Therefore, replication in adolescents from other countries and cultural settings is warranted.

Our findings should not be interpreted as evidence for a biological dose–response relationship or sweetener-specific associations. Rather, frequent ZCB consumption may serve as a behavioral marker associated with depressive symptoms and related psychosocial or lifestyle factors. If confirmed in longitudinal studies, this readily ascertainable dietary behavior may help identify adolescents who could benefit from further assessment of their mental health and related health behaviors. Nevertheless, the present findings should be interpreted cautiously given the cross-sectional design and should not be considered evidence of a causal relationship or as a basis for recommendations regarding ZCB consumption for the prevention of depressive symptoms.

## 5. Conclusions

Higher ZCB consumption was associated with depressive symptoms among Korean adolescents, with a stronger observed association among females. Given the cross-sectional design, these findings should be interpreted as evidence of an observed association that may reflect broader psychosocial and lifestyle factors rather than a causal effect of ZCB consumption. As ZCB use continues to increase among adolescents, prospective longitudinal studies incorporating quantitative dietary assessment, biomarker-supported exposure measures, and validated diagnostic assessment of depression are needed to clarify temporal relationships and the biological and psychosocial mechanisms underlying these associations.

## Figures and Tables

**Table 1:** Baseline characteristics of study participants by zero-calorie beverage consumption frequency (*n* = 50,972).

Variable	Zero-Calorie Beverage Consumption Frequency				
	None	≤2/Week	3–6/Week	≥1/Day	Total
N (%)	28,669 (56.2)	19,138 (37.5)	2564 (5.0)	601 (1.2)	50,972 (100.0)
Age category, years					
12–13	7872 (27.5)	5110 (26.7)	493 (19.2)	127 (21.1)	13,602 (26.7)
14–15	10,093 (35.2)	6384 (33.4)	792 (30.9)	196 (32.6)	17,465 (34.3)
16–18	10,704 (37.3)	7644 (39.9)	1279 (49.9)	278 (46.3)	19,905 (39.1)
Sex					
Male	13,636 (47.6)	10,292 (53.8)	1636 (63.8)	399 (66.4)	25,963 (50.9)
Female	15,033 (52.4)	8846 (46.2)	928 (36.2)	202 (33.6)	25,009 (49.1)
BMI, kg/m^2^ (mean ± SD)	21.09 ± 3.63	21.96 ± 3.81	22.81 ± 4.10	22.91 ± 4.41	21.53 ± 3.77
BMI category ^a^					
Underweight	1610 (5.6)	605 (3.2)	75 (2.9)	24 (4.0)	2314 (4.5)
Normal weight	20,994 (73.2)	13,242 (69.2)	1662 (64.8)	376 (62.6)	36,274 (71.2)
Overweight or obese	6065 (21.2)	5291 (27.6)	827 (32.3)	201 (33.4)	12,384 (24.3)
Household economic status					
High	11,965 (41.7)	8078 (42.2)	1158 (45.2)	278 (46.3)	21,479 (42.1)
Middle	13,634 (47.6)	8942 (46.7)	1112 (43.4)	238 (39.6)	23,926 (46.9)
Low	3070 (10.7)	2118 (11.1)	294 (11.5)	85 (14.1)	5567 (10.9)
School type					
Middle school	15,749 (54.9)	10,054 (52.5)	1096 (42.7)	289 (48.1)	27,188 (53.3)
High school	12,920 (45.1)	9084 (47.5)	1468 (57.3)	312 (51.9)	23,784 (46.7)
Academic performance					
High	11,125 (38.8)	7004 (36.6)	908 (35.4)	162 (27.0)	19,199 (37.7)
Middle	8256 (28.8)	5678 (29.7)	738 (28.8)	158 (26.3)	14,830 (29.1)
Low	9288 (32.4)	6456 (33.7)	918 (35.8)	281 (46.8)	16,943 (33.2)
Ever smoker ^b^	1841 (6.4)	1649 (8.6)	335 (13.1)	94 (15.6)	3919 (7.7)
Ever drinker ^c^	6942 (24.2)	5529 (28.9)	953 (37.2)	239 (39.8)	13,663 (26.8)
SSB frequency					
<1/week	13,478 (47.0)	7108 (37.1)	534 (20.8)	148 (24.6)	21,268 (41.7)
Weekly	13,999 (48.8)	11,319 (59.1)	1752 (68.3)	148 (24.6)	27,218 (53.4)
Daily	1192 (4.2)	711 (3.7)	278 (10.8)	305 (50.7)	2486 (4.9)
High-caffeine beverage frequency					
<1/week	24,073 (84.0)	14,734 (77.0)	1472 (57.4)	312 (51.9)	40,591 (79.6)
Weekly	4087 (14.3)	4061 (21.2)	944 (36.8)	143 (23.8)	9235 (18.1)
Daily	509 (1.8)	343 (1.8)	148 (5.8)	146 (24.3)	1146 (2.2)
Daily smartphone use, min (mean ± SD)	302.2 ± 169.3	308.8 ± 168.2	330.9 ± 181.6	389.6 ± 230.7	307.1 ± 170.8
Physical activity ^d^					
Low (0–2 d/wk)	18,582 (64.8)	11,138 (58.2)	1341 (52.3)	303 (50.4)	31,364 (61.5)
Moderate (3–4 d/wk)	5719 (19.9)	4428 (23.1)	596 (23.2)	132 (22.0)	10,875 (21.3)
High (≥5 d/wk)	4368 (15.2)	3572 (18.7)	627 (24.5)	166 (27.6)	8733 (17.1)
Breakfast skipper ^e^	12,274 (42.8)	8505 (44.4)	1187 (46.3)	284 (47.3)	22,250 (43.7)
Depressive symptoms	6905 (24.1)	5062 (26.4)	758 (29.6)	229 (38.1)	12,954 (25.4)

^a^ BMI category was defined using sex- and age-specific percentiles from the 2017 Korean National Growth Charts: underweight (<5th percentile), normal weight (5th to <85th percentile), and overweight or obese (≥85th percentile). Observations with BMI < 10 or >50 kg/m^2^ were excluded as implausible. ^b^ Ever smoker = lifetime use of any tobacco product (conventional cigarettes, e-cigarettes, or heated tobacco products). ^c^ Ever drinker = any lifetime alcohol use beyond a small curiosity sip. ^d^ Physical activity categorized according to the WHO adolescent guideline based on number of days per week with ≥60 min of moderate-to-vigorous physical activity. ^e^ Breakfast skipper = breakfast consumption ≤ 2 days per week. Values are presented as unweighted counts. All regression analyses incorporated the complex survey design and sampling weights. Abbreviations: BMI, body mass index; SD, standard deviation; SSB, sugar-sweetened beverage.

**Table 2:** Association between zero-calorie beverage consumption and depressive symptoms among Korean adolescents (*n* = 50,972).

Zero-Calorie Beverage Consumption	Model 1	Model 2	Model 3
	Adjusted OR (95% CI) ^a^	Adjusted OR (95% CI) ^b^	Adjusted OR (95% CI) ^c^
None	1.00 (Ref)	1.00 (Ref)	1.00 (Ref)
≤2 times/week	1.17 (1.11–1.22)	1.13 (1.07–1.18)	1.10 (1.05–1.16)
3–6 times/week	1.42 (1.29–1.57)	1.28 (1.16–1.42)	1.14 (1.03–1.27)
≥1 time/day	2.01 (1.67–2.42)	1.76 (1.45–2.13)	1.32 (1.08–1.62)
*p* for trend	<0.001	<0.001	<0.001

^a^ Model 1: adjusted for sex, age category, school type, household economic status, and academic performance. ^b^ Model 2 (primary): adjusted for age category, school type, household economic status, academic performance, BMI category, lifetime smoking, lifetime alcohol use, physical activity, breakfast skipping, and daily smartphone use (tertile). ^c^ Model 3: Model 2 plus sugar-sweetened and high-caffeine beverage frequency. *p* for trend was tested by entering the four-category exposure as an ordinal variable. Abbreviations: CI, confidence interval; OR, odds ratio.

**Table 3:** Sex-stratified association between zero-calorie beverage consumption and depressive symptoms among Korean adolescents (*n* = 50,972).

Zero-Calorie Beverage Consumption	Male, Adjusted OR (95% CI)			Female, Adjusted OR (95% CI)		
	Model 2 ^a^	Model 3 ^b^		Model 2 ^a^		Model 3 ^b^
None	1.00 (Ref)	1.00 (Ref)		1.00 (Ref)		1.00 (Ref)
≤2 times/week	1.09 (1.01–1.16)	1.07 (0.99–1.14)		1.16 (1.09–1.23)		1.12 (1.05–1.20)
3–6 times/week	1.06 (0.92–1.22)	0.97 (0.84–1.12)		1.65 (1.42–1.93)		1.43 (1.22–1.67)
≥1 time/day	1.63 (1.28–2.07)	1.32 (1.03–1.70)		2.07 (1.54–2.79)		1.41 (1.02–1.95)
*p* for trend	0.001	0.085		<0.001		<0.001
*p* for interaction ^c^			<0.001			

^a^ Model 2 (primary): adjusted for age category, school type, household economic status, academic performance, BMI category, lifetime smoking, lifetime alcohol use, physical activity, breakfast skipping, and daily smartphone use (tertile). ^b^ Model 3: Model 2 plus sugar-sweetened and high-caffeine beverage frequency. ^c^ From a single survey-weighted model fitted to both sexes with a 3-df zero-calorie beverage × sex product term (*F* (3, 681) = 8.66). *p* for trend was tested by entering the four-category exposure as an ordinal variable. Abbreviations: CI, confidence interval; OR, odds ratio.

## Data Availability

The KYRBS data analyzed in this study are publicly available from the Korea Disease Control and Prevention Agency (https://www.kdca.go.kr/yhs/) (accessed on 17 May 2026).

## Supplementary Materials

The following supporting information can be downloaded at: https://www.mdpi.com/article/10.3390/nu18162610/s1, Figure S1. Flow diagram of participant selection from the 2025 Korea Youth Risk Behavior Web-based Survey (KYRBS). Of 54,170 eligible adolescents aged 12–18 years, 3198 were excluded because of missing data on one or more study variables, yielding a final analytic sample of 50,972 participants (25,963 males and 25,009 females). Figure S2. Directed acyclic graph (DAG) illustrating the assumed causal structure relating zero-calorie beverage (ZCB) consumption to depressive symptoms. The bold arrow indicates the association of interest (ZCB consumption → depressive symptoms). Solid arrows represent assumed causal relationships underlying confounding pathways, and dashed arrows indicate potential residual confounding from unmeasured factors. The primary model (Model 2) adjusted for measured confounders considered common causes of both ZCB consumption and depressive symptoms, including sex, age, school type, household economic status, academic performance, body mass index, lifetime smoking, lifetime alcohol use, physical activity, breakfast skipping, and daily smartphone use. Sugar-sweetened and high-caffeine beverages were additionally included in Model 3 as potential confounders and/or markers of dietary and lifestyle patterns. Because their causal roles cannot be determined in a cross-sectional study, Model 3 was considered a sensitivity analysis, recognizing the possibility of residual confounding or over-adjustment if these variables lie on the causal pathway; Table S1. Model-standardized predicted prevalences, prevalence differences, prevalence ratios, and additive interaction for the association between zero-calorie beverage consumption and depressive symptoms according to sex (*n* = 50,972); Table S2. Sex-specific association between zero-calorie beverage consumption and depressive symptoms under alternative exposure coding (*n* = 50,972); Table S3. Sex-stratified association between zero-calorie beverage consumption and depressive symptoms after collapsing the two highest consumption categories (*n* = 50,972).

## References

[1] Miller L., Campo J.V. (2021). Depression in Adolescents. N. Engl. J. Med..

[2] Thapar A., Eyre O., Patel V., Brent D. (2022). Depression in young people. Lancet.

[3] Shorey S., Ng E.D., Wong C.H.J. (2022). Global prevalence of depression and elevated depressive symptoms among adolescents: A systematic review and meta-analysis. Br. J. Clin. Psychol..

[4] Zhu F., Yang Y., Yin T., Pan M., Xu J., Chen R., Zheng W., Gu F. (2025). The Burden of adolescent depression and the impact of COVID-19 across 204 countries and regions from 1990 to 2021: Results from the 2021 global burden of disease study. Sci. Rep..

[5] Barth C., Crestol A., de Lange A.M.G., Galea L.A.M. (2023). Sex steroids and the female brain across the lifespan: Insights into risk of depression and Alzheimer’s disease. Lancet Diabetes Endocrinol..

[6] Copeland W.E., Worthman C., Shanahan L., Costello E.J., Angold A. (2019). Early Pubertal Timing and Testosterone Associated With Higher Levels of Adolescent Depression in Girls. J. Am. Acad. Child. Adolesc. Psychiatry.

[7] Ladouceur C.D. (2026). Sex Differences in Affective Disorders: A Developmental Neuroscience Framework on the Role of Puberty. Annu. Rev. Clin. Psychol..

[8] Kim Y., Park H., Bhan Y., Lee D., Oh C.M., Lee W.Y., Park B. (2025). Changes in Mental Health Among Adolescents in South Korea Before and After COVID-19: An Interrupted Time Series Analysis from 2015 to 2022. J. Adolesc. Health.

[9] Woo H.G., Park S., Yon H., Lee S.W., Koyanagi A., Jacob L., Smith L., Cho W., Min C., Lee J. (2023). National Trends in Sadness, Suicidality, and COVID-19 Pandemic-Related Risk Factors Among South Korean Adolescents from 2005 to 2021. JAMA Netw. Open.

[10] Jung S., Lee E.H.L., Kim J.Y., Park S., Lee J.E., Ultra-Processed Food Working Group (2026). Trends in Ultraprocessed Food Consumption Among Korean Children and Adolescents, 2007 to 2024. JAMA Netw. Open.

[11] Xie J., Huang Z., Mo Y., Pan Y., Ruan Y., Cao W., Chen Y., Li Y., Li K., Yu D. (2025). Ages-specific beverage consumption and its association with depression and anxiety disorders: A prospective cohort study in 188,355 participants. J. Affect. Disord..

[12] Akbaraly T., Norton J., Mura T., Jaussent I., Lassale C., Jacka F.N., O’Neil A., Kivimaki M., Ashtree D.N. (2026). Exposure to Global Burden of Disease dietary factors and risk of recurrent depressive symptoms: The prospective Whitehall II cohort study. Eur. J. Nutr..

[13] Guo X., Park Y., Freedman N.D., Sinha R., Hollenbeck A.R., Blair A., Chen H. (2014). Sweetened beverages, coffee, and tea and depression risk among older US adults. PLoS ONE.

[14] Samuthpongtorn C., Nguyen L.H., Okereke O.I., Wang D.D., Song M., Chan A.T., Mehta R.S. (2023). Consumption of Ultraprocessed Food and Risk of Depression. JAMA Netw. Open.

[15] Klein S.D., Collins P.F., Wun V.L., Grund P., Luciana M. (2025). Frontostriatal Networks Undergo Functional Specialization during Adolescence That Follows a Ventral-Dorsal Gradient: Developmental Trajectories and Longitudinal Associations. J. Neurosci..

[16] Walker D.M., Bell M.R., Flores C., Gulley J.M., Willing J., Paul M.J. (2017). Adolescence and Reward: Making Sense of Neural and Behavioral Changes Amid the Chaos. J. Neurosci..

[17] Ren Z., Gao L., Hao Z., Zhao B., Li J., Li G., Cao J., Tang X. (2025). Association between dietary diversity and risk of depressive symptoms in Chinese children, adolescents, and college students. Front. Nutr..

[18] Ulm C., Hill F., Mills S.D., Chelius C., Hecht E.M. (2026). Association between unhealthy food consumption and mental health outcomes in children and adolescents: An umbrella review. Child. Adolesc. Psychiatry Ment. Health.

[19] Korea Disease Control and Prevention Agency The Korea Youth Risk Behavior Web-Based Survey (KYRBS). https://www.kdca.go.kr/yhs/.

[20] Kim Y., Choi S., Chun C., Park S., Khang Y.H., Oh K. (2016). Data Resource Profile: The Korea Youth Risk Behavior Web-based Survey (KYRBS). Int. J. Epidemiol..

[21] Verlenden J., Pampati S., Viox M.H., Brener N., Licitis L., Dittus P., Ethier K. (2024). Measuring Population-Level Adolescent Mental Health Using a Single-Item Indicator of Experiences of Sadness and Hopelessness: Cross-Sectional Study. JMIR Form. Res..

[22] Kim J.H., Yun S., Hwang S.S., Shim J.O., Chae H.W., Lee Y.J., Lee J.H., Kim S.C., Lim D., Yang S.W. (2018). The 2017 Korean National Growth Charts for children and adolescents: Development, improvement, and prospects. Korean J. Pediatr..

[23] de Onis M., Onyango A.W., Borghi E., Siyam A., Nishida C., Siekmann J. (2007). Development of a WHO growth reference for school-aged children and adolescents. Bull. World Health Organ..

[24] Bull F.C., Al-Ansari S.S., Biddle S., Borodulin K., Buman M.P., Cardon G., Carty C., Chaput J.P., Chastin S., Chou R. (2020). World Health Organization 2020 guidelines on physical activity and sedentary behaviour. Br. J. Sports Med..

[25] Andersson T., Alfredsson L., Kallberg H., Zdravkovic S., Ahlbom A. (2005). Calculating measures of biological interaction. Eur. J. Epidemiol..

[26] Knol M.J., VanderWeele T.J. (2012). Recommendations for presenting analyses of effect modification and interaction. Int. J. Epidemiol..

[27] Di K.N., Chi V.T., Duc T.Q. (2026). Soft drink consumption associated with depressive symptoms among the general population: Consistent and robust evidence from a systematic review and meta-analysis. Food Funct..

[28] Liu B.P., Jia C.X., Li S.X. (2022). Soft drink consumption and depressive symptoms among the adolescents of United States: The mediating role of aggressive behaviors. J. Affect. Disord..

[29] Zhang Z., Li W., Zhang M., Zhang Y. (2025). High sugar-sweetened beverage intake predicts adverse physical, emotional, and sleep health trajectories in adolescents: A 4-year prospective cohort study. Front. Public Health.

[30] Sahril N., Adnan M.A.A., Khalil M.K.N., Chan Y.M., Ratnam K.K.Y., Lai W.K., Ahmad N.A. (2023). Association of dietary behaviour and depression among adolescents in Malaysia: A cross-sectional study. J. Health Popul. Nutr..

[31] Thanarajah S.E., Ribeiro A.H., Lee J., Winter N.R., Stein F., Lippert R.N., Hanssen R., Schiweck C., Fehse L., Bloemendaal M. (2025). Soft Drink Consumption and Depression Mediated by Gut Microbiome Alterations. JAMA Psychiatry.

[32] Sailesh S.S. (2026). Association between soft drink consumption and depression mediated by gut microbiome: A perspective. East Asian Arch. Psychiatry.

[33] Ahmed S.P., Pi-Sunyer B.P., Moses-Payne M.E., Goddings A.L., Speyer L.G., Kuyken W., Dalgleish T., Blakemore S.J. (2024). The role of self-referential and social processing in the relationship between pubertal status and difficulties in mental health and emotion regulation in adolescent girls in the UK. Dev. Sci..

[34] Andersen E., Klusmann H., Eisenlohr-Moul T., Baresich K., Girdler S. (2024). Life stress influences the relationship between sex hormone fluctuation and affective symptoms in peripubertal female adolescents. Dev. Psychopathol..

[35] Cruz-Carrillo G., Herrejon K., Ruiz-Gonzalez R., Roque A., Zamudio-Flores J., Lajud N. (2026). Mild fructose diet during periadolescent development increases emotionality, but not metabolic consequences of early life adversity in rats. J. Dev. Orig. Health Dis..

[36] Santerre-Anderson J.L., Colon M.S., Pike B.L., Kiessling C.Y. (2025). Adolescent high-fructose corn syrup consumption contributes to protracted dysregulation of adult accumbal neuroinflammation and affective behaviors. Brain Res..

[37] Pereira M.A. (2013). Diet beverages and the risk of obesity, diabetes, and cardiovascular disease: A review of the evidence. Nutr. Rev..

[38] Vanselow M.S., Pereira M.A., Neumark-Sztainer D., Raatz S.K. (2009). Adolescent beverage habits and changes in weight over time: Findings from Project EAT. Am. J. Clin. Nutr..

[39] Solmi F., Sharpe D.H., Gage S.H., Maddock J., Lewis G., Patalay P. (2021). Changes in the Prevalence and Correlates of Weight-Control Behaviors and Weight Perception in Adolescents in the UK, 1986–2015. JAMA Pediatr..

[40] Rosenbaum J.E. (2009). Truth or consequences: The intertemporal consistency of adolescent self-report on the Youth Risk Behavior Survey. Am. J. Epidemiol..

[41] Brener N.D., Kann L., Shanklin S., Kinchen S., Eaton D.K., Hawkins J., Flint K.H., Centers for Disease Control and Prevention (CDC) (2013). Methodology of the Youth Risk Behavior Surveillance System—2013. MMWR Recomm. Rep..

[42] Copeland K.T., Checkoway H., McMichael A.J., Holbrook R.H. (1977). Bias due to misclassification in the estimation of relative risk. Am. J. Epidemiol..

[43] Chatelan A., Lebacq T., Rouche M., Kelly C., Fismen A.S., Kalman M., Dzielska A., Castetbon K. (2022). Long-term trends in the consumption of sugary and diet soft drinks among adolescents: A cross-national survey in 21 European countries. Eur. J. Nutr..

[44] Sylvetsky A.C., Jin Y., Clark E.J., Welsh J.A., Rother K.I., Talegawkar S.A. (2017). Consumption of Low-Calorie Sweeteners among Children and Adults in the United States. J. Acad. Nutr. Diet..

[45] Hwang S.B., Park S., Jin G.R., Jung J.H., Park H.J., Lee S.H., Shin S., Lee B.H. (2020). Trends in Beverage Consumption and Related Demographic Factors and Obesity among Korean Children and Adolescents. Nutrients.

