# Supplementary material for: Sex-Specific Association Between Zero-Calorie Beverage Consumption and Depressive Symptoms in Korean Adolescents: A Nationwide Cross-Sectional Study
Source: Nutrients. 2026 Aug 10;18(16):2610. doi: 10.3390/nu18162610 (PMC13517050; doi:10.3390/nu18162610)
Supplement: Supplementary file 1 [file nutrients-18-02610-s001.zip › Supplementary_Tables_major revision.pdf]

Supplementary Table S1. Model-standardized predicted prevalences, prevalence differences, prevalence ratios, and additive interaction for the association between zero-calorie beverage consumption and depressive symptoms according to sex (N = 50,972)

**Zero-calorie beverage consumption**	**Male**			**Female**		
	**Predicted prevalence (%)**	**Difference in predicted prevalence, percentage points (95% CI)**	**Prevalence ratio (95% CI)**	**Predicted prevalence (%)**	**Difference in predicted prevalence, percentage points (95% CI)**	**Prevalence ratio (95% CI)**
None (Ref)	20.8	Ref	1.00 (Ref)	28.0	Ref	1.00 (Ref)
≤2 times/week	22.0	+1.2 (0.0 to 2.3)	1.06 (1.00–1.11)	31.1	+3.1 (1.9 to 4.4)	1.11 (1.06–1.16)
3–6 times/week	21.2	+0.5 (−1.8 to 2.7)	1.02 (0.91–1.13)	39.2	+11.2 (7.8 to 14.6)	1.40 (1.28–1.53)
≥1 time/day	28.7	+7.9 (3.1 to 12.7)	1.38 (1.15–1.61)	44.5	+16.6 (9.6 to 23.5)	1.59 (1.34–1.84)
Additive interaction (female sex × ≥1 /day)[a]	Difference in prevalence differences, +8.7 percentage points (95% CI, 0.5 to 16.9); RERI, 0.42 (95% CI, 0.02 to 0.81); P = 0.038					

Estimates were obtained from a survey-weighted logistic model including both sexes with a zero-calorie beverage × sex interaction and Model 2 covariates, accounting for the complex sampling design. Predicted prevalences were estimated as predictive margins within each sex. Prevalence differences and ratios were derived from these estimates using the delta method.

[a] RERI was calculated using male/None as the reference cell. A positive value indicates supra-additive interaction, and the null was excluded, suggesting that the absolute excess prevalence associated with daily consumption was greater in females and males.

Abbreviations: CI, confidence interval; RERI, relative excess risk due to interaction.

Supplementary Table S2. Sex-specific association between zero-calorie beverage consumption and depressive symptoms under alternative exposure coding (N = 50,972)

Zero-calorie beverage consumption	Male, OR (95% CI)		Female, OR (95% CI)
**Categorical (indicator)— primary** [a]			
None	1.00 (Ref)		1.00 (Ref)
≤2 times/week	1.09 (1.01–1.16)		1.16 (1.09–1.23)
3–6 times/week	1.06 (0.92–1.22)		1.65 (1.42–1.93)
≥1 time/day	1.63 (1.28–2.07)		2.07 (1.54–2.79)
P for trend	0.001		<0.001
P for sex interaction (joint, 3 df) [b]		<0.001	
**Approximate servings-per-week score** [c]			
Overall, per 1 additional serving/week		1.07 (1.05–1.09)	
Per 1 additional serving/week [a]	1.04 (1.02–1.06)		1.12 (1.09–1.15)
P for sex interaction (1 df) [d]		<0.001	

All models were survey-weighted and adjusted as in Model 2 (age category, school type, household economic status, academic performance, body mass index category, lifetime smoking, lifetime alcohol use, physical activity, breakfast skipping, and daily smartphone use tertile), accounting for the complex sampling design.

[a] The categorical (indicator) coding is the primary analysis and reproduces the Model 2 estimates in Table 3; it is shown here for direct comparison. Sex-specific estimates are from sex-stratified models, which exclude sex.

[b] Joint test of the three exposure × sex product terms from a single model fitted to both sexes combined (adjusted Wald test, $F(3, 681) = 8.66$).

[c] Each category was assigned an approximate number of servings per week (none = 0, ≤2/week = 1, 3–6/week = 4.5, and ≥1/day = 7); because the highest category is open-ended, it was assigned its lower bound. The overall estimate is adjusted for sex and includes no interaction term.

[d] Test of the exposure × sex product term from a single model fitted to both sexes combined (adjusted Wald test, $F(1, 683) = 24.12$).

Abbreviations: CI, confidence interval; OR, odds ratio.

Supplementary Table S3. Sex-stratified association between zero-calorie beverage consumption and depressive symptoms after collapsing the two highest consumption categories (N = 50,972)

Zero-calorie beverage consumption	Unweighted n (male / female)	Male, adjusted OR (95% CI)		Female, adjusted OR (95% CI)	
		**Model 2[a]**	**Model 3[b]**	**Model 2[a]**	**Model 3[b]**
None	13,636 / 15,033	1.00 (Ref)	1.00 (Ref)	1.00 (Ref)	1.00 (Ref)
≤2 times/week	10,292 / 8,846	1.09 (1.01–1.16)	1.07 (1.00–1.14)	1.16 (1.09–1.23)	1.12 (1.05–1.20)
≥3 times/week	2,035 / 1,130	1.17 (1.03–1.32)	1.04 (0.92–1.17)	1.72 (1.50–1.97)	1.43 (1.23–1.65)
P for trend[c]		0.003	0.150	<0.001	<0.001
P for sex interaction[d]		<0.001		<0.001	

[a] Model 2 (primary), adjusted as in Table 3; sex-stratified models exclude sex. [b] Model 3: Model 2 plus sugar-sweetened and high-caffeine beverage frequency.

[c] P for trend was tested by entering the three-category exposure as an ordinal variable within each sex. [d] Joint 2-df test of the collapsed exposure × sex terms from a single model fitted to both sexes (adjusted Wald test: Model 2, $F(2, 682) = 11.86$; Model 3, $F(2, 682) = 11.41$).

The two highest categories were combined because the daily-consumption category was sparse (n = 601 overall; 202 female adolescents). Consumption of at least three times per week remained associated with depressive symptoms and the sex interaction was preserved, indicating that the findings did not depend on this category.

Abbreviations: CI, confidence interval; OR, odds ratio.
